# Gene rearrangement analysis and ancestral order inference from chloroplast genomes with inverted repeat

**DOI:** 10.1186/1471-2164-9-S1-S25

**Published:** 2008-03-20

**Authors:** Feng Yue, Liying Cui, Claude W dePamphilis, Bernard ME Moret, Jijun Tang

**Affiliations:** 1 Department of Computer Science and Engineering, University of South Carolina, Columbia, SC 29208, USA; 2 Department of Biology, Pennsylvania State University, University Park, PA 16802, USA; 3 School of Computer and Communication Sciences, Swiss Federal Institute of Technology (EPFL), EPFL IC LCBB, INJ 230, Station 14, CH-1015 Lausanne, Switzerland; 4 Swiss Institute of Bioinformatics

## Abstract

**Background:**

Genome evolution is shaped not only by nucleotide substitutions, but also by structural changes including gene and genome duplications, insertions, deletions and gene order rearrangements. The most popular methods for reconstructing phylogeny from genome rearrangements include GRAPPA and MGR. However these methods are limited to cases where equal gene content or few deletions can be assumed. Since conserved duplicated regions are present in many chloroplast genomes, the inference of inverted repeats is needed in chloroplast phylogeny analysis and ancestral genome reconstruction.

**Results:**

We extend GRAPPA and develop a new method GRAPPA-IR to handle chloroplast genomes. A test of GRAPPA-IR using divergent chloroplast genomes from land plants and green algae recovers the phylogeny congruent with prior studies, while analysis that do not consider IR structure fail to obtain the accepted topology. Our extensive simulation study also confirms that GRAPPA has better accuracy then the existing methods.

**Conclusions:**

Tests on a biological and simulated dataset show GRAPPA-IR can accurately recover the genome phylogeny as well as ancestral gene orders. Close analysis of the ancestral genome structure suggests that genome rearrangement in chloroplasts is probably limited by inverted repeats with a conserved core region. In addition, the boundaries of inverted repeats are hot spots for gene duplications or deletions. The new GRAPPA-IR is available from .

## Background

**Figure 1:**
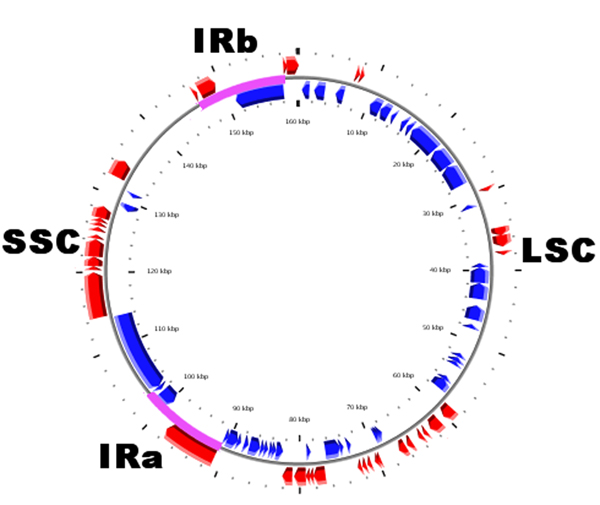
**Gene map of the Gossypium hirsutum chloroplast genome**. The bright thick lines indicate the extent of the inverted repeats (IRa and IRb), which separate the genome into small (SSC) and large (LSC) single copy regions. The gene map is drawn using CGView [35].

Mutations in a genome consist of not only base pair level changes but also events that alter the chromosome structure, such as inversions, duplications or deletions [1]. Ancestral gene sequence inference has led to significant predictions of protein functional shift and positive selection [2]. For example, comparisons of orthologous chromosomal segments showed heterogeneous rates of evolution of the X chromosome in human, mouse and rat [3]. However, on the genome level, the evolutionary change of genome structure is poorly understood. Inference of ancestral genomes was mainly achieved at the DNA level, but limited to closely related organisms where rearrangements were negligible, partly because of the complexity in assigning genes in duplicated segments to orthologous groups [4]. In this paper, we take a simple, alternative data set of chloroplast genomes to study the phylogeny and genome structural changes. Chloroplasts are the green, photosynthetic organelles that originated from a free-living cyanobacteria-like ancestor [5]. Chloroplasts maintained a reduced genome through over one billion years of endosymbiosis [6]. Typical chloroplast genomes are circular single chromosomes with 120 — 200 genes, which encode proteins, tRNAs, rRNAs and hypothetical open reading frames. Most chloroplast genomes consist of four distinct parts: two duplicated regions (inverted repeats, or *IR*) separated by a large single copy *(SSC)* and a small single copy *(ISC)* region. Figure 1 shows the four regions (LSC-IRa-SSC-IRb) of *Gossypium hirsutum* chloroplast [7]. One common characteristic of the chloroplast IR is the presence of three rRNA genes *(rrn5s, rrn16s* and *rrn23s,* or *rrf, rrs,* and *rrl*), which are homologous to genes of the cyanobacterial* rrn* operon. The structure of chloroplast genomes of land plants is highly conserved, with almost collinear gene order, except for elevated level of rearrangements in specific lineages including green algae [8], conifers and members of a few flowering plant families including *Campanulaceae *[9], *Geraniaceae*[10] and *Fabaceae*[11]. The gene content of chloroplast IRs vary greatly, largely due to the expansion and contraction of the IR at the IR-SC boundaries; this "ebb and flow" of IR boundary has been observed even within a genus [12,13]. Chloroplast genomes of green algae (charophyte and chlorophyte algae) also contain more variations of gene order and some are highly rearranged [8], with some evidence that rearrangements may be adaptive in nature [37]. Because of their compact size and the availability of conserved DNA probes, many chloroplast genomes have been mapped [14], and 121 have been completely sequenced to date. Thus, chloroplast genomes provide an ideal example for modeling genome rearrangements over a broad evolutionary time scale.

### Genome rearrangements

We assume a reference set of *n* genes {*g_1_, g_2_,* • • •, *g_n_*}, thus a genome can be represented as a signed ordering of these genes, and each gene is given an orientation that is either positive, written *g_i_*, or negative, written —*g_i_.* Genomes can evolve through events such as inversions, transpositions and transversion, as well as other events such as deletions, insertions and duplications of fragments. Let *G* be the genome with signed ordering of *g1, g2,*…, *g_n_*. An *inversion* (reversal) between indices *i* and *j (i ≤ j),* transforms *G* to a new genome with linear ordering

$$\;g_{1},\;g_{2},\cdots ,\;g_{i-1},-g_{j},-g_{j-1},\cdots ,-g_{j},g_{j+1},\;\cdots ,\;g_{n}$$

A *transposition* on genome *G* acts on three indices *i,j,k,* with *i* ≤ *j* and *k* &owm; [*i,j*], picking up the interval *gi*,*gi*+1, … ,*gj* and inserting it immediately after *gk*. Thus genome *G* is replaced by (assume *k* > *j*):

$$\;g_{1},\cdots g_{i-1},g_{j+1},\cdots g_{k},g_{i},g_{i+1},\cdots ,g_{j},g_{k+1},\;\cdots ,g_{n}$$

Because gene rearrangements are *rare genomic events*[15], gene-order data enable the reconstruction of evolutionary events far back in time, thus many biologists have embraced this new source of data in their phylogenetic work [9,14,16,38].

### Genome rearrangement analysis

Given two genomes *G_1_* and *G_2_,* we define the *edit distance d(G_1_,* G_2_) as the minimum number of events required to transform one genome into the other. The *breakpoint distance *[17] is not a direct evolutionary distance measurement. A breakpoint in *G_1_* is defined as an ordered pair of genes *(g_i_, g_j_)* such that *g_i_* and *g_j_* are adjacent in *G_1_* but not in *G_2_.* The breakpoint distance is simply the number of breakpoints in *G_1_* relative to *G_2_.* When only inversions are allowed, the edit distance is the *inversion distance.* Hannenhalli and Pevzner [18] developed a mathematical and computational framework for signed gene-orders and provided a polynomial-time algorithm to compute the edit distance between two signed gene-orders under inversions; Bader et al. [19] later showed that this edit distance can be computed in linear time. However, computing the inversion distance is NP-hard in the unsigned case [20].

Gene rearrangement phylogeny was first proposed by Sankoff and an algorithm using break-point distance was implemented in BPAnalysis [4]. The inversion phylogeny was introduced to improve the accuracy and was implemented in GRAPPA. Extensive simulations showed that inversion median were superior to breakpoint median [21] and the trees returned were more accurate than using either distance-based or parsimony methods [22]. The current version of GRAPPA (version 2.0) is able to estimate the phylogeny and inversion medians using genomes with equal gene content (i.e., no insertion, deletion or duplication are allowed) [22]. A scaled-up version, DCM-GRAPPA, is able to estimate the gene-order phylogeny with very high accuracy for thousands of genomes, thus greatly increasing the power of genome phylogeny using large datasets [23]. We extended GRAPPA [24] so that it is able to analyze data sets with a limited number of deletions, but no duplication is allowed. To remedy this problem, we develop a new algorithm (GRAPPA-IR) for chloroplast genomes that take into account the unique quadripartite structure (e.g., LSC-IRa-SSC-IRb), which is common to not only the chloroplast genomes, but also some other IR-containing DNAs. The assumption of our new approach is that inversions do not occur across inverted repeats, because the genome structure will be disrupted by such inversions that "flip" the repeats from inverted to the same orientation. According to the model, a change of gene content within the IR region is mainly due to growth or shrinkage of the IR at the IR-SC boundaries. This approach is in agreement with the observation that in most IR-containing chloroplasts, the gene content in the whole genome is conserved, but IR length and IR gene contents can be varied.

## Results and discussion

We assess the new GRAPPA-IR through both biological and simulated datasets.

### Analysis of six chloroplast genomes

We select a test case of six chloroplast genomes representing major lineages of land plants and green algae, all of which share the quadripartite structure (LSC-IRa-SSC-IRb). The organisms include *Nicotiana tobacum* (tobacco, *nt), Psilotum nudum* (whisk fern, *pn), Marchantia polymorpha* (liverwort, *mp*), *Chaetosphaeridium globosum* (a charophyte alga, *cp), Nephroselmis olivacea* (a chlorophyte alga, *no*), and *Mesostigma uiride* (a photosynthetic protist, *mv*).

**Figure 2:**
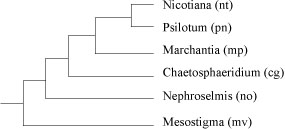
**The reference phylogeny of chloroplast genomes from land plants and green algae**.

**Figure 3:**
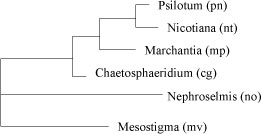
**The best tree obtained by GRAPPA-IR. The topology is the same as the reference tree**.

A reference phylogenetic tree was constructed using the maximum parsimony method with 50 concatenated proteins. Cyanophora proteins were used to root the tree. The reference phylogeny of these six chloroplast genomes is shown in Figure 2. The reference tree is the same as the phylogeny by Lemieux et al. [25] in which *Mesostigma* is basal to other green plants and algae. An alternative phylogeny was published by Karol et al. based on maximum likelihood analysis of four chloroplast genes and including many more algal taxa, in which *Mesostigma* is basal to charophyte green algae and sister to chlorophyte green algae [26]. We extracted 73 unique genes from the six genomes. Actual number of genes included in each genome ranges from 76 to 80 due to duplicated genes in AIR. The gene set includes 62 characterized protein-coding genes, 3 rRNAs, 7 tRNAs (identified by amino acid anticodons) and a hypothetical conserved open reading frame *(ycf1).* The encoding reflects the order and orientation of genes in the genome. Location of multi-exon genes was determined by the starting position of the first exon. In one case, the order of overlapping genes *(psbD-psbC)* was determined by the position of the start codon. We evaluate all possible trees for the six genomes using the GRAPPA-IR. After 100 min of computation on a PIV 3.4GHz workstation, GRAPPA-AIR returned a best tree with 74 inversions, with a topology agrees with the reference tree, which is shown in Figure 3.

**Figure 4:**
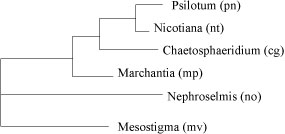
**The best tree when no IR boundary is imposed, with score 73**. Notice that the topology is different from the reference tree.

We tested this data set with the original GRAPPA by ignoring the region boundaries and removing the IRb region. The inference allows inversions to occur across the IR and single copy regions. The best tree obtained requires 73 inversions, yet the topology (Figure 4) is very different from the reference trees and is in conflict with the biological relationship of these organisms. Although GRAPPA is a heuristic, extensive testing on simulated and biological data confirmed its high accuracy, thus its failure in this test suggests that the IR-boundary do perform a unique role in the evolution of chloroplast genomes, and a better method as GRAPPA-IR should be preferred.

We examined the reconstructed ancestral gene orders on the best tree returned by GRAPPA-IR. This six-genome dataset contains chloroplast genomes of land plants, green algae and a flagellate protist, which were separated by at least 450 million years of evolution [27]. The ancestral chloroplast genomes of land plants and algae contain inverted repeats, which is consistent with the hypothesis that IR is a feature derived early in the chloroplast endosymbiosis [16]. Although the sequenced cyanobacteria *Nostoc* and *Synechococcus* do not maintain rrn-containing IRs, if other cyanobacteria are identified with structures similar to the chloroplast IR, then it would suggest an even earlier origin for this structure. In addition, the ancestral IR contains the same gene content to that of *Mesostigma,* which agrees with the observation that *Mesostigma* chloroplast genome encodes several ancestral gene clusters [25]. By comparison of ancestral gene orders to the extant genomes, it is possible to test formally the evolutionary force of gene order changes. For example, ancestral gene clusters may be more likely to be maintained if they share related function and are under constraints in the face of genome rearrangements.

### Simulations to assess accuracy

Phylogenetic analysis methods deal with lost historic information, thus their accuracy should also be assessed through simulations, where the true evolutionary history is known.

We first define our measure for the accuracy of reconstructed trees. Given an inferred tree, we compare its "topological accuracy" by computing "false negatives" and "false positives" with respect to the "true tree" [28]. For every tree there is a natural association between every edge and the bipartition on the leaf set induced by deleting the edge from the tree.

Let *T* be the true tree and let *T'* be the inferred tree. An edge e in *T* is "missing" in *T'* if T' does not contain an edge defining the same bipartition; such an edge is called a *false negative* (FN). Note that the external edges (i.e. edges incident to a leaf) are trivial in the sense that they are present in every tree with the same set of leaves. The *false negative rate* is the number of false negative edges in *T'* with respect to *T* divided by the number of internal edges in *T.* The *false positive (FP) rate* is defined similarly, by swapping *T* and *T'.* The *Robinson-Foulds* (RF) rate is defined as the average of the FN and FP rates.

In this study, we generated datasets of 6 and 10 genomes, each with 78 genes (70 genes in LSC, 5 in SSC and 3 in IR), roughly in the range of our dataset described in the paper. We used a large range of evolutionary rates: let *r* denote the expected number of evolutionary events along an edge of the model tree, we used values of *r* in the range of 4—10. The actual number of inversions along each edge is sampled from a uniform distribution on the set {1,2,…, 2r}. Given the model tree, we assigned the identity gene order to the root, and randomly generated gene order for each node based on the edge length and the gene order of its parent, with the assumption that inversions can not cross the IR boundaries. For each combination of parameter settings, we simulated 20 datasets and averaged the results.

**Figure 5:**
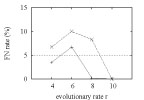
**FN rate for GRAPPA-IR (solid line) and GRAPPA (dashed line) as a function of the evolutionary rate *r* for 6 genomes**. The horizontal line indicates the 5% error level, a typical threshold of acceptability for accurate phylogenetic reconstruction [36].

**Figure 6:**
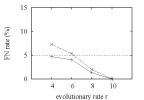
**FN rate for GRAPPA-IR (solid line) and GRAPPA (dashed line) as a function of the evolutionary rate *r* for 10 genomes**.

We compared GRAPPA-IR to the original GRAPPA. We considered all trees with the minimum score given by both methods and took their strict consensus (of course, most time there is only one single best tree was returned). Therefore, the trees returned by both methods need not to be fully resolved and they tend to have somewhat better rates for false positives than for false negatives. As a result, we only report FN rates rather than FP rates or a single Robinson-Foulds score [29]. Figure 5 and Figure 6 show the results. This simulation indicates that GRAPPA-IR is clearly more accurate than the original GRAPPA for datasets with *r* < 10, which is in accordance with results on the six-genome dataset.

Since the inferred ancestral genomes have direct impact on the tree scores, we can examine the quality of the inferred ancestral genomes by comparing the best tree score returned by GRAPPA-IR with the known true tree score. Averagely speaking, scores of the best trees returned by GRAPPA-IR was only about 7% less than the true scores, which strongly indicates that the inferred ancestral gene orders should be very close to the true ancestral genomes.

## Discussion

### Mechanisms of IR expansion

The gene content of the IR varies across land plants, even in a single genus or family [12]. It is known that homologous recombination is frequent between the two copies of IR [30]. In a single chloroplast, hundreds of copies of chloroplast DNA co-exist as circular monomer, dimer and linear chromosomes. In the cellular endosymbiosis environment, the selection on accuracy of replication may have been relaxed to the degree that unequal recombination and replication slippage contribute to the expansion or shrinkage of IRs. Short repeat motifs may facilitate inter-molecular recombination and create diversity of chloroplast genomes in a population [31]. On the other hand, the intra-molecular recombination process should homogenize the sequences of the two IRs and thus the particular IR size and the gene content are maintained. The two counteracting phenomena may have played important roles in shaping the current diversity of chloroplast genome gene orders.

### Duplications and genome stability

We found that incorrect gene order phylogenies were recovered without considering the IR boundary information. This suggests that maintenance of the IR is necessary in the evolution of chloroplast genomes in most of the cases. We propose that IR provides an insulation mechanism that stabilizes the genome structure, and the genes in single copy regions do not commute across the IR. This agrees with the observation that gene rearrangements are more frequent in chloroplast genomes without IR [32]. However, some genomes with residual IRs but infrequent gene movements between single copy regions compared to related lineages do not conform to the hypothesis [9]. Future experimental studies on highly rearranged chloroplast genomes, for example, in the green alga Chlamydomonas lineage [8,37], may shed light on the maintenance of IR and genome rearrangements.

### Comparison to other methods

A similar approach used for human and mouse genome comparison showed the optimal sorting of X chromosomes by at least 7 inversions [33]. This is a moderate amount of change compared to the level we observe in many chloroplast genomes. If duplications and deletions are considered in a finer scale, the process will be much complex, as suggested by the reconstruction of one 1.1 Mb region in the eutherian mammal ancestor [4]. All methods proposed in this paper could be applied to MGR and other method, which will result in a whole new set of tools for botanists interested in genome level evolution.

## Conclusions

We implement a new method to infer phylogeny and ancestral gene orders with inverted repeats. Tests on a biological and simulated dataset show GRAPPA-IR can accurately recover the genome phylogeny as well as ancestral gene orders. Close analysis of the ancestral genome structure suggests that genome rearrangement in chloroplasts is probably limited by inverted repeats with a conserved core region. In addition, the boundaries of inverted repeats are hot spots for gene duplications or deletions. This analysis provides new insight into the genome evolutionary process.

## Methods

We developed a new method (GRAPPA-IR) to handle inverted repeats. This method is available online from .

### Mapping contents in ancestral genomes

The first step of our new method-GRAPPA-IR is to determine the gene contents for each region in the genomes involved. Based on our previous research [24], such mapping can dramatically reduce the search space and improve the overall accuracy. When a genome is on a leaf (i.e., it is an extant taxon), we can easily determine the gene content for the LSC, ISC and IR regions through direct observation. However, since we do not know the gene order at each internal genome, we can only estimate the gene content for each region based on the assumption that all evolutionary events that alter the gene order are rare and that concurrent (i.e., parallel) changes in two children are less likely than a change in the parent. Thus, at each internal node, for a given region, when the regional gene contents for the two children are known, we face three possibilities of assigning a gene to the region:

1. If both children have gene *g* in the same region, then the parent has *g* in that region; otherwise, both children need to expand (or shrink) IRs and include g in that region, with a very low probability.

2. If neither child has *g,* then *g* is absent in the parent. Since the genomes we test all share 70 unique genes, we do not consider this case.

3. If *g* is located in different regions between the children, then it could be in either region of the parent. The two choices are equally likely without further information from the phylogeny. If the tree is rooted, we use the gene content in the evolutionary path to break the tie; otherwise, we are left with an undetermined outcome for *g.*

**Figure 7:**
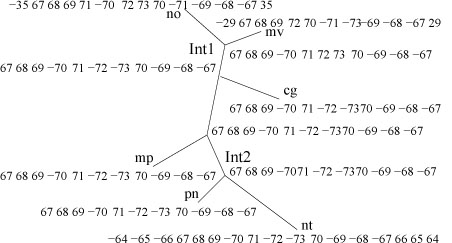
**Estimated gene contents for each region (only IR and SSC are shown)**.

If a gene is undetermined in some internal node, it may become determined through a propagation of decisions from the leaves to the root. The estimated gene content for the internal nodes of the reference tree is presented in Figure 7. This figure shows that the gene contents of the IR and ISC regions vary among the genomes. However the gene order of part of IR is highly conserved. For example, some genes (rrn5, rrn16, rrn23, coded as 67, 68 and 69, respectively) are always kept together in the IR. If we assume that inversions do not cross the IR boundary in most chloroplast genomes, then the evolution of chloroplast genome structure can be hypothesize as undergoing the following two steps:

1. A genome was divided into regions and inversions occurred independently in each region.

2. A segment from single copy regions was copied twice and joined to the existing inverted repeats, and the new genomes with longer IRs propagated.

**Figure 8:**
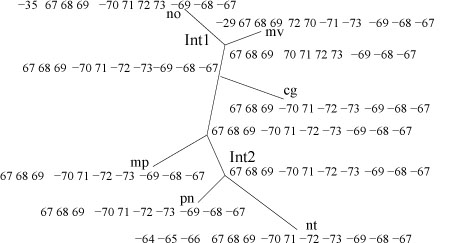
**Revised estimation of gene contents for each region (only IR and SSC are shown)**.

One should notice that the above two steps could happen several times along each edge. Based on the above assumption, we can further simplify the gene content of IR and SSC, so that in the evolutionary path, IR regions for all genomes (leaves and internal) contain gene (67 68 69), and the SSC regions contain gene (70 71 72 73). This operation treats duplicate genes at the boundaries of IR and SSC as the last step towards the observed gene orders in the evolutionary path. Thus it is possible to ignore the duplications and reduce the problem to all leaf genomes of equal gene content (or with deletions). The simplified gene content map is shown in Figure 8.

### Phylogeny and ancestral genome reconstruction

We can reconstruct the phylogeny after the regional gene contents of all genomes are determined. Since the gene contents are reduced to equal after the simplification step, it is feasible to use GRAPPA to infer an inversion phylogeny. That's to say, if inversions are allowed to cross the boundaries of pre-determined IR and single copy regions, we can use the original GRAPPA to compute the ancestral gene orders and the phylogeny. However, this is unlikely, since we do not observe any inversions involving genes in both the IR and single copy regions. Based on our proposed model, we assume that all inversions are bounded by the boundary of IRs.

Bounding inversions into each region makes each region (*LSC, SSC* and *IR*) independent, which greatly simplify the computation. For two genomes *G_1_* and *G_2_*, the genomic distance between these two can be defined as *d*(*G*_1_,*G*_2_) = *d*(*SSC*_1_, *SSC*_2_) + *d*(*LSC*_1_, *LSC*_2_) + *d*(*IR*_1_, *IR*_2_), i.e., the overall distance is the summation of all regional distances. If no gene content is changed for each region, then the regional distance can be computed using the linear algorithm proposed by [19]. However, if the regional content is not equal (deletions or insertions occur), then more complex algorithm [34] should be used. The smallest binary tree contains only three leaf genomes and one ancestor. This special case is called *median problem* in some literatures. Given three genomes *G*_1_, *G*_2_ and *G*_3_, solving a median problem is to find a genome *G*_0_ that can minimize the sum of distances from itself to the three given genomes. Although it is the simplest case for multiple genome analysis, it is proved to be NP hard even for the simplest distance of breakpoint. Since we can deal with each region independently, the median problem can also be divided into three regional median problems, each of which is constructed from genes in the same region of genomes *g*_1_, *g*_2_ and *g*_3_. After the regional median is obtained, the median solution on the whole genome can be constructed simply by concatenating the regions together. Again, the regional median problems can be solved using any of the available inversion median solvers, such as [20] (for equal regional gene content) or [24] (with deletions and insertions).

**Figure 9:**
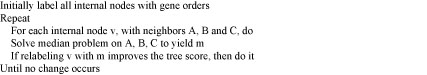
**The scoring procedure of GRAPPA-IR when the tree is given**.

To analyze a dataset with more than three genomes, GRAPPA-IR uses an exhaustive approach devised for the original GRAPPA-it must test all possible trees to find the one with the minimum number of inversions. For each tree, the program tests a lower bound [22] to determine whether the tree is worth scoring; if so, then the program will iteratively solve the median problems at internal nodes and update the internal genomes, until no change occurs, as outlined in Figure 9. The tree with the lowest score (smallest number of changes) will be returned as the phylogeny, and internal gene orders with respect to the phylogeny can be treated as the estimated ancestral genomes.

## Competing interests

The authors declare that they have no competing interests.

## Authors' contributions

L.C. and C.D. contributed to the data preparation, data analysis, and draft of the manuscript. F.Y., B.M. and J.T. contributed to the development and implementation of the algorithms. F.Y. and J.T. were in charge of the simulation studies.  L.C. and J.T. wrote the initial draft of the manuscript with contributions from F.Y., B.M., and C.D.  All authors read and approved the final manuscript.
